# Supplementary material for: The interactome of intact mitochondria by cross-linking mass spectrometry provides evidence for coexisting respiratory supercomplexes
Source: Mol Cell Proteomics. 2017 Dec 8;17(2):216–32. doi: 10.1074/mcp.RA117.000470 (PMC5795388; doi:10.1074/mcp.RA117.000470)
Supplement: Supplemental Data [file supp_RA117.000470_133922_0_supp_23978_fzffwf.zip › spectra_annotation/mito_DR_spectra_annotation/304-1-1-1-8-1.pdf]

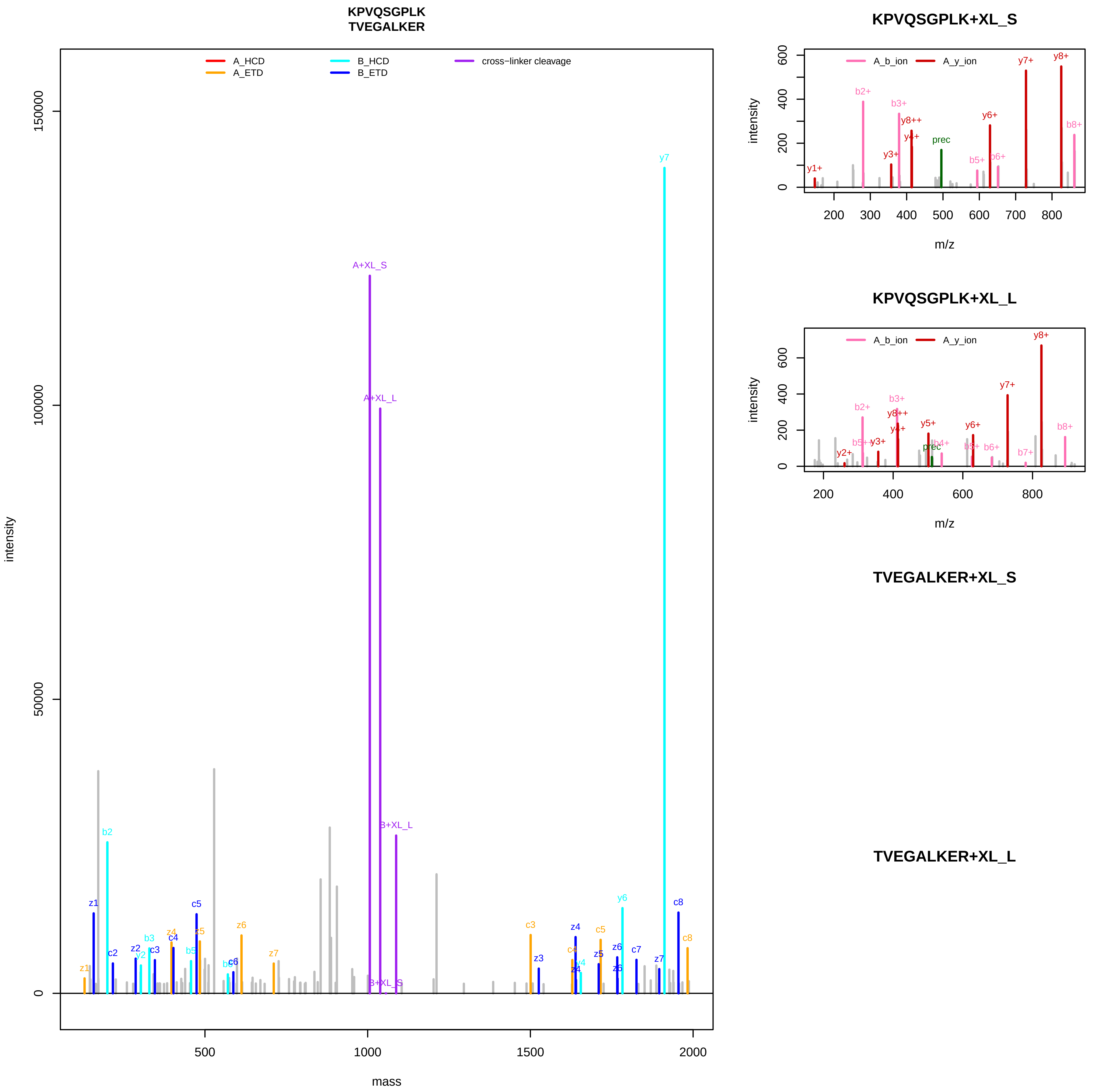

KPVQSGPLK
TVEGALKER
A_HCD
A_ETD
B_HCD
B_ETD
cross−linker cleavage
intensity
mass
0
50000
100000
150000
500
1000
1500
2000
A+XL_S
A+XL_L
B+XL_L
B+XL_S
y7
y6
b2
b3
b5
z1
c2
z2
y2
c3
c4
z4
z5
c5
c6
z6
z7
c3
z3
c4
z4
y4
z5
c5
z6
c7
z7
c8
KPVQSGPLK+XL_S
A_b_ion
A_y_ion
intensity
m/z
y1+
b2+
b3+
y3+
y8++
y4+
prec
b5+
y6+
b6+
y7+
y8+
b8+
KPVQSGPLK+XL_L
A_b_ion
A_y_ion
intensity
m/z
y2+
b2+
b3+
y8++
y3+
y4+
y5+
prec
b4+
y6+
b6+
b7+
y7+
y8+
b8+
TVEGALKER+XL_S
TVEGALKER+XL_L
